# Five Successful Cases of General Suppurative Peritonitis Treated by a New Method*Read before the Medical and Chirurgical Faculty of the State of Maryland at Its Annual Meeting in Baltimore, April 27, 1897.

**Published:** 1897-09

**Authors:** J. M. T. Finney

**Affiliations:** Associate Professor of Surgery, The Johns Hopkins University


### FIVE SUCCESSFUL CASES OF GENERAL SUPPURATIVE PERITONITIS TREATED BY A NEW METHOD.*

BY J. M. T. FINNEY, M. D.,

Associate Professor of Surgery, The Johns Hopkins University.

Recovery following laparotomy for purulent peritonitis is unfortunately of sufficient rarity to excite interest whenever it occurs. My object in making this report to the Society is two-fold; first, to record five successful cases of laparatomy for general suppurative peritonitis, all treated by the same method; and second, to describe briefly the method itself. The principal involved is not a new one; only in the manner of carrying it out is there any originality claimed.

Since the appearance in 1877 of the classical work of Wegner, and later that of Grawitz and others, it has been known that the healthy peritoneum is capable of disposing of a considerable amount of infectious material. J. G. Clark, in a recent article,† reviews the literature of the subject and gives the conclusions reached by the experimenters in this direction. All agree that the peritoneum is able under favorable conditions to take up a relatively large amount of infectious material and dispose of it effectually. These observers were dealing with a more or less healthy peritoneum. On opening the abdomen of a patient suffering from general suppurative peritonitis, however, we have very different conditions with which to deal. The observations of Pawlowsky would indicate that the lymph channels leading from the peritoneal cavity are choked with infectious bacteria and inflammatory products in purulent peritonitis, and that thus the efficiency of the peritoneum would be greatly impaired. Our observations clinically seemed hardly to bear this out.

The question that suggested itself to our mind was this, whether or not the peritoneum, even under these most unfavorable conditions, still retained it absorptive power. It seemed to us, from our experience in operating upon such cases by the methods heretofore employed, that they were inadequate and did not remove a sufficient quantity of the exudate, but left

---

*Read before the Medical and Chirurgical Faculty of the State of Maryland at its Annual Meeting in Baltimore, April 27, 1897.

†Bulletin of The Johns Hopkins Hospital, April, 1897.

the peritoneum little bettter off than before. With this idea in mind we devised a plan of treatment which, so far as we know, has not been employed elsewhere.

The steps of the operation are as follows: Make a sufficiently long incision to admit of easy access to all parts of the peritoneal cavity. Quickly withdraw the coils of small intestine from the peritoneal cavity, beginning with the worst coils first. Remove all, or as much as is necessary of the small intestine and place it outside the abdomen, covered with warm gauze or towels, thus practically disemboweling the patient for the time being. Then thoroughly and systematically wipe out the peritoneal cavity with large pledgets of gauze wrung out of hot salt solution, paying particular attention to the pelvic portion. In some cases it may be well in addition to flush out the cavity with warm salt solution, but this is rarely necessary.

Next the small intestine should be systematically examined, loop by loop, while still outside the abdomen, and rendered macroscopically clean by wiping with gauze compresses wrung out of hot salt solution. It is necessary to wipe with considerable force at times, in order to remove adherent flakes of partly organized lymph. It should be done thoroughly and conscientiously, however, as upon this depends, we believe, in great measure, the success of the operation. It facilitates the cleansing process, as well as lessens the shock of the operation, if the wiping of the intestinal coils is carried on under a constant irrigation of warm salt solution.

After being cleansed macroscopically of all foreign material, pus, feces, lymph, etc., the intestine should be replaced in the abdomen—the worst or sutured coil being the last, or most superficial, in order that it may be the better drained by being packed about with gauze, if necessary.

The abdominal wound is then tightly closed, leaving just room enough between two sutures for the gauze drain. If there are any evidences of distention or pain the abdomen should have the Paquelin cautery thoroughly applied, and the bowels moved early by calomel in broken doses, followed by salts and a turpentine enema.

It is not claimed for this method that it will cure every case of general suppurative peritonitis. We believe, however, that a larger percentage of cases will recover after this method than any other with which we are familiar.

To insure success with any method it is essential that the operation should be performed within a few hours after the perforation has taken place. This is well brought out in the very interesting series of experiments on dogs made for me by Messrs. Elting and Calvert of the Johns Hopkins Medical School, a report of which is subjoined.

Five cases have been reported upon by this method up to date, all of which have recovered. The first case, a case of perforating typhoid ulcer, has already been published,‡ and hence, only a very brief abstract of the history will be given here.

CASE I.—Male, aged 47, on about eighth day of mild attack of typhoid developed symptoms of perforation. Entered hospital fourteen hours later and was operated upon immediately. Peritoneum everywhere intensely congested, roughened and dull, and covered with flakes of plastic lymph. Considerable amount of turbid purulent fluid in abdominal cavity. Perforation in ileum about fourteen inches from ileo-cæcal valve. Fecal matter exuding from opening. Peritoneum cleansed in the manner described, gauze drainage. Recovery.

CASE II.—G. W., male, aged 20. Saw patient for the first time, November 24, 1896, in consultation with Dr. Barringer in Charlottesville, Va. Patient gave history of four previous mild attacks of appendicitis, from which he had promptly recovered. The night before he had eaten very heartily of apples. He was awakened about 3 A. M. with severe abdominal pain, cramp-like in character. At about 6 A. M. Dr. Barringer was called. He stated that at this time, three hours after the beginning of the attack, the patient presented the classical symptoms of peritonitis. When I saw him, twenty-four hours later, he had a temperature of 102 degrees and a pulse of over 100, and from the first had suffered intense pain, which was controlled only by morphia hypodermically. He had had nausea and vomiting all day. Examination of the abdomen showed slight distention and great rigidity of the abdominal muscles. A slight tumefaction could be made out just to the inner side of the anterior superior spine of the ilium on the right side. Tenderness very marked. Immediate operation advised and agreed to. Incision five inches long in right linea semilunaris. On opening the peritoneal cavity the intestinal coils in the right lower quadrant of the abdomen were found to be congested and dull and covered with flakes of adherent lymph. Elsewhere the intestinal coils were found to be congested, but not otherwise much changed in appearance. The pus, of which there was perhaps 200cc., was not walled off, but everywhere present in pockets between the adherent intestinal coils. The appendix was readily found. It was closely adherent to the pelvic brim on one side and the cæcum on the other. Its distal end was swollen and distended to the size of my thumb, perforated and gangrenous over an area about as large as a five-cent piece. Appendix was ligated and excised, and stump covered with peritoneal cuff and suture. The peritoneum was treated in the manner above described. Recovery.

‡Annals of Surgery, March, 1897.

CASE III.—This patient was seen first on December 14, 1896. His history is in brief as follows: R. S., male, aged 33 years. Has had no previous similar attack. The night before he was taken sick he attended a banquet and ate heartily of solid, indigestible food. He was attacked with severe abdominal pain about 3 o'clock the next afternoon. The pain at first was general and cramp-like; nausea and light vomiting during the night. Morphia was necessary to relieve him. The next day he was unable to get up. Toward evening his physician gave him a cathartic, after which the bowels moved eight or ten times in quick succession. The next morning the pain had shifted to the right side and was severe. He received a hypodermic of morphia and got on fairly well until about 6 P. M., about sixty hours after the onset of the attack, when he was taken with a sudden, severe pain in the lower right side of the abdomen. The pain for a time was excruciating at the base of the penis. Vesical and rectal tenesmus marked. When I saw him, about four hours later, in consultation with Dr. Reiche, he had a temperature of 105 degrees and pulse of 150, profoundly collapsed. I have never seen such a hard and retracted abdomen as he presented. His condition appeared grave. Immediate operation advised and consented to.

Incision about five inches long, in right linea semilunaris. On opening abdomen the intestinal coils were found not to be distended but considerably congested. Beginning in the right lower quadrant there was found a considerable amount of thin pus containing flakes of lymph. This condition extended over into the left side, down into the pelvis, and up into the hypogastric region. The appendix was found to be gangrenous and perforated, and was removed. The toilet of the peritoneum was made in the manner already described, by disemboweling and vigorously scrubbing the parietal and visceral peritoneum until macroscopically clean. The intestinal coils were then replaced, a gauze drain inserted, and the abdominal wound closed except a small opening for the drain. He made an uninterrupted recovery.

CASE IV.—M. B., boy, aged 10. Operation by Dr. J. C. Bloodgood, January 7, 1897. Five days before admission to the hospital was struck in the abdomen by the fist of a playmate. Next day felt severe pain in the right iliac region. This progressively increased for three days, when vomiting began and the pain became general. Two days later was brought to the hospital, when his condition was found to be in brief as follows: Temperature 101 degrees, pulse 128 and fairly good. Slight abdominal distention. Muscular spasm marked on right side, present, but less marked, on the left. General abdominal tenderness, Under either a definite tumefaction could be made out in the region of the right kidney. This proved to be an abscess behind the cæcum, extending from the iliac fossa below to the liver above, and in this cavity was the diseased appendix. There was found no walling off of this from the general peritoneal cavity. The entire pelvis was filled with yellow pus and all the intestinal coils were covered with flakes of fibrin. The stomach and spleen were not seen, but the surface of the liver looked exactly as if it had been covered with yellowish-white paint. The appendix was removed and the entire abdominal cavity thoroughly wiped out with gauze pledgets wrung out of salt solution. The exudate was scrubbed off the liver's surface, after which it looked simply congested. A gauze drain was inserted and the abdominal wound partly closed. He made an uninterrupted recovery. Cultures and cover-slips from the peritoneum showed colon bacillus and a coccus (not differentiated).

CASE V.—R. S. P., aged 9, a schoolboy, entered the Johns Hopkins Hospital, Feb. 26, 1897. He had always been healthy except for measles, whooping-cough, and chicken-pox.

Family history good except remote cases of tuberculosis on both sides.

Just forty-eight hours before entering the hospital first complained of pain in abdomen. Three hours later had an attack of vomiting. Pain in abdomen was at first general, but in a few hours became localized in the right iliac and lumbar regions. After about twenty-four hours the pain lessened somewhat, and he sat up for a little while, but shortly after pain and vomiting returned with increased severity. A physician saw him after about thirty-six hours and gave him calomel in broken doses. His bowels moved twice. His condition did not improve, and by advice of his physician was brought to the hospital at 8 P. M., forty-eight hours after the onset of the attack. His condition then was as follows: Face flushed and anxious. Pulse 126; temp. 102.8 degrees; resp. 56, and entirely thoracic; abdomen generally distended and tender especially in right iliac fossa, where the tenderness is extreme and muscle spasm very marked. Pain is most marked here also. Liver and spleen not palpable. Liver dullness on right corresponds about to costal border. Percussion over right iliac and lumbar regions shows dullness; tympanitic over left side. Heart normal. Fine moist rales over bases of both lungs. No history of any similar previous attack.

### DIAGNOSIS.

Perforating appendicitis with beginning general peritonitis. Immediate laparotomy advised and agreed to. Ether. When thoroughly anæsthetized, a small, hard mass, somewhat movable, could be felt just over the middle of Poupart's ligament. An incision about 15 cm. long was made parallel to and over the right linea semilunaris. After exposing the peritoneum and before opening it several bubbles of gas could be seen free in the peritoneal cavity. On opening the peritoneum a considerable amount of thin, cloudy sero-purulent fluid escaped and some gas. The mass felt before was found to be the appendix with a roll of omentum adherent. The intestines, especially the cæcum, were distended and congested, and covered with flakes of fresh fibrinous exudate. The congestion was most marked in the immediate vicinity of the appendix.

The appendix itself was superficially placed and freely movable, not walled off, but had a portion of omentum adherent. It was rather long, and curled upon itself, with a constriction at about the junction of its proximal and middle thirds. It contained two concretions, the larger of which was engaged tightly in the constriction, and from this point to the tip the appendix was gangrenous and softened. A small perforation was present at the distal end of the date-seed like concretion. There had been an apparent attempt of the omentum to surround the entire gangrenous end of the appendix, but it had not quite succeeded. The appendix, together with the adherent omentum was ligated and excised.

Pelvis was found to be full of pus, and the peritoneum treated as above. He made a rapid and complete recovery.

Bacteriological examination of the peritoneal exudate showed the presence of streptococcus, staphylococcus, and bacillus coli communis.

NOTE.—Since reading the above article, I have operated upon one additional case of general peritonitis. The patient, a young woman, was *in extremis* at the time of the operation, which was undertaken simply as a forlorn hope. This operation was secondary to one performed several days previously by another surgeon for appendicular abscess. There was found present a general peritonitis, with much plastic lymph covering the greatly distended adherent coils of intestine. There was very little purulent fluid in the abdomen. Her pulse was very rapid and thready, and her temperature had risen several degrees. After the operation she was placed in a continuous bath, which added greatly to her comfort. The operation seemed to prolong her life, as she lived about thirty-six hours following it.—*Johns Hopkins Hospital Bulletin.*

